# Pregabalin Effect on Acute and Chronic Pain after Cardiac Surgery

**DOI:** 10.1155/2017/2753962

**Published:** 2017-04-30

**Authors:** Aik Bouzia, Vassilios Tassoudis, Menelaos Karanikolas, George Vretzakis, Argyro Petsiti, Nikolaos Tsilimingas, Elena Arnaoutoglou

**Affiliations:** ^1^Intensive Care Unit, Medical School of Larissa, University of Thessaly, Volos, Greece; ^2^Department of Anesthesiology, Medical School of Larissa, University of Thessaly, Volos, Greece; ^3^Department of Anesthesiology, Washington University School of Medicine, St. Louis, MO, USA; ^4^Department of Cardiothoracic Surgery, Medical School of Larissa, University of Thessaly, Volos, Greece

## Abstract

*Introduction.* Pain after cardiac surgery affects long-term patient wellness. This study investigated the effect of preoperative pregabalin on acute and chronic pain after elective cardiac surgery with median sternotomy.* Methods.* Prospective double blind study. 93 cardiac surgery patients were randomly assigned into three groups: Group 1 received placebo, Group 2 received oral pregabalin 75 mg, and Group 3 received oral pregabalin 150 mg. Data were collected 8 hours, 24 hours, and 3 months postoperatively.* Results.* Patients receiving pregabalin required fewer morphine boluses (10 in controls versus 6 in Group 1 versus 4 in Group 2, *p* = 0.000) and had lower pain scores at 8 hours (4 versus 3 versus 3, *p* = 0.001) and 3 months (3 versus 2 versus 2, *p* = 0.000) and lower morphine consumption at 8 hours (14 versus 13 versus 12 mg, *p* = 0.000) and 24 hours (19.5 versus 16 versus 15 mg, *p* = 0.000). Percentage of patients with sleep disturbances or requiring analgesics was lower in the pregabalin group and even lower with higher pregabalin dose (16/31 versus 5/31 versus 3/31, *p* = 0.000, and 26/31 versus 16/31 versus 10/31, *p* = 0.000, resp.) 3 months after surgery.* Conclusion.* Preoperative oral pregabalin 75 or 150 mg reduces postoperative morphine requirements and acute and chronic pain after cardiac surgery.

## 1. Introduction

Acute postsurgical pain and the possible progression to chronic postsurgical pain (CPSP) can influence patients' immediate and late postoperative course after cardiac surgery [1]. Sources of pain after cardiac surgery with median sternotomy include the sternotomy incision, chest tubes, the pericardial drainage, the site of saphenous vein, or radial artery harvesting [2, 3]. The pain is described as chest discomfort of noncardiac origin in up to 65% of cases and can coexist with pain in the upper extremities, neck, head, and midback area [4]. Acute postoperative pain after coronary artery bypass grafting (CABG) surgery adversely affects pulmonary function in the first two postoperative days and can potentially delay extubation and prolong hospital stay [5, 6], thereby adversely affecting patient recovery. Furthermore, acute pain can progress to chronic pain with hypoesthesia and allodynia along the surgical incision. 56% of patients describe sternotomy pain as pain of medium intensity that affects daily activities, while 38% of patients report unbearable pain. Chronic pain after cardiac surgery can adversely influence quality of life even a year after surgery [7–10], with one-third of patients with chronic pain reporting sleep disturbances. In patients over 60 years of age, pain has been recorded even 28 months after surgery with reported frequency as high as 40%. Studies in female patients showed that pain persisted over a year with a significant percentage requiring treatment by a physician or physiotherapist [10].

Approaches used in an attempt to reduce pain after cardiac surgery include local anesthetic infiltration of the sternotomy and chest tube insertion sites [11], parasternal block [12], thoracic epidural analgesia [13, 14], and analgesic medications, including intravenous opioids [15, 16], intrathecal opioids [17], and paracetamol, diclofenac, and gabapentin by different routes [18–20].

Pregabalin (Lyrica, Pfizer, Inc.) is a beta-isobutyl of GABA with chemical similarity to gabapentin [21]. Pregabalin binds to the alpha-2-delta subgroup of calcium channels, thereby reducing excitatory neurotransmitter release and preventing hyperalgesia and central sensitization [22]. Compared to gabapentin, pregabalin seems to have more potent analgesic effects with fewer adverse effects [21]. Pregabalin is used as anticonvulsant but has also been used as analgesic for neuropathic pain and, lately, for postoperative pain, in an attempt to reduce opioid consumption and prevent progression to chronic pain [23–25].

Pregabalin may have a role in controlling acute postoperative pain and published evidence suggests that it may be effective in prevention of chronic postsurgical pain (CPSP) [24, 26, 27]. In addition, there is evidence that pregabalin confers an opioid-sparing effect in patients undergoing cardiac surgery [28]. Therefore, it is plausible that pregabalin could reduce acute pain and opioid requirements after cardiac surgery and may help prevent the transition from acute to chronic pain after cardiac surgery. This study was conducted to investigate the impact of a single preoperative oral pregabalin dose on morphine consumption in the immediate postoperative period and on the transition from acute to chronic pain in patients undergoing elective cardiac surgery with median sternotomy.

## 2. Methods

After approval by the Ethics Committee, 93 patients scheduled for cardiac surgery in a tertiary care University Hospital enrolled in this double blind, placebo controlled, randomized study. The study protocol was in agreement with the Helsinki Declaration on patient safety during anesthesiology research [29] and was registered in the “ClinicalTrials.gov” clinical trial registration website (ClinicalTrials.gov identifier: NCT01701921).

Inclusion criteria were elective primary cardiac surgery with median sternotomy and extracorporeal circulation, ages 18–85 years, and written informed consent for participation in the study. Exclusion criteria included previous cardiac or thoracic surgery, allergy to pregabalin, previous use of gabapentin or pregabalin, chronic pain, known diagnosis of depression or other major psychiatric diseases, cognitive impairment or inability to cooperate with the study, renal insufficiency, and history of substance abuse. Patient demographic and clinical data were stored in a secure, encrypted electronic database. Based on a computer custom number generator, patients were randomly assigned to one of three groups: Group 1 = control (patients received a placebo capsule), Group 2 = low dose pregabalin (patients received a capsule containing 75 mg of pregabalin) (Lyrica, Pfizer Ltd.), and patients in Group 3 = high dose pregabalin received a capsule containing 150 mg of pregabalin. The study drug (pregabalin or placebo) was given to patients by a research coordinator and was documented in the medical record as “study drug.” All anesthesia personnel taking care of patients in the operating room were blinded to group assignment.

After introduction of a large intravenous catheter in the upper extremity and placement of standard monitoring (ECG, noninvasive blood pressure, pulse oximetry, and capnography) the radial artery was cannulated under local anesthesia. Then, a bispectral index (BIS) sensor was placed, general anesthesia was induced using etomidate 0.3 mg/kg and fentanyl 30 mcg/kg, and intubation was facilitated with vecuronium 0.1 mg/kg. Anesthesia was initially maintained using sevoflurane at 1–1.2 age-adjusted MAC until insertion of a cordis in the right internal jugular vein and placement of a continuous cardiac output/mixed venous oximetry pulmonary artery catheter. Then, after central line placement was completed, anesthesia was maintained using propofol 100–200 *μ*g/kg/minute and remifentanil infusion 0.1–0.2 *μ*g/kg/min, titrated to maintain BIS values in the 35 to 50 range, and sevoflurane was discontinued. All operations were performed by the same surgeon, through midline sternotomy, using cardiopulmonary bypass with a roller pump and membrane oxygenator; pump flow rate was approximately 2.5 L/min/m^2^ in order to maintain perfusion pressure around 50 mm Hg. During cardiopulmonary bypass, core temperature was normal for coronary artery bypass graft (CABG) procedures, while mild hypothermia was applied for valve replacement operations. At the end of surgery, patients remained intubated and were transported to the ICU under continuing propofol and remifentanil infusion for sedation. Criteria for weaning from the ventilator and extubation were hemodynamic stability, absence of excessive chest tube drainage or arrhythmias, normothermia, adequate urine output, spontaneous ventilatory frequency < 20 breaths/min, and a cooperative patient that could respond to basic commands. After extubation, when patient condition was satisfactory, patients received a single 5 mg dose of intravenous morphine and patient control analgesia (PCA) was started with a programmable PCA pump (Gemstar, Abbott) using the following settings: basal morphine infusion 0.5 mg/hour, bolus dose 0.5 mg, and lockout time 30 minutes. Analgesia was supplemented with IV paracetamol 1 gm every 8 hours for the first 24 hours after surgery. In addition, if the analgesic regimen was not sufficient despite maximum PCA use, we advised ICU clinicians to provide additional (rescue) analgesia with IV morphine as deemed necessary. The goal was to keep pain scores measured by Verbal Rating Scale (VRS) (0 = no pain to 10 = unbearable pain) at rest ≤ 4 for every patient. An anesthesiologist blinded to group assignment visited the patients in 8 hours and 24 hours after extubation, recorded VRS after a deep breath, documented the presence and severity of nausea and vomiting, and collected data on PCA use and morphine consumption from the Gemstar pump. In addition, a blinded searcher interviewed all patients by telephone 3 months after surgery, inquired about the impact of the operation on their lives, and asked specific questions about the presence and severity of pain after surgery. In cases where patients indicated they were still experiencing pain, the researcher also collected data about use of analgesics and the presence of sleep disorders.

### 2.1. Statistical Analysis

Sample size calculation was based on assumptions supported by the findings of an earlier pilot, unpublished study, as follows: primary outcome is morphine consumption at 24 hours, morphine consumption at 24 hours is mean = 20 mg, with SD = 5 mg, and a change of morphine use by 4 mg is a clinically meaningful change. Using these assumptions, the required sample size to have alpha error = 0.05 and power = 0.8 is 26 patients per group. Based on this calculation, we decided to increase sample size by 20% to 32 patients per group, in order to reduce the risk of not having adequate sample size due to patient attrition, missing data, errors in patient allocation, or other study shortcomings.

Normality of continuous data was assessed with the Kolmogorov-Smirnov and Shapiro-Wilk tests, and findings regarding normality were visually validated with normal Q-to-Q plots and detrended normal Q-to-Q plots. Based on this analysis, data on age, weight, height, BMI, and operation times were analyzed using ANOVA, whereas all other continuous variables were analyzed using the nonparametric Kruskal-Wallis test. When ANOVA or Kruskal-Wallis test showed a significant difference between groups for a particular variable, Student's* t*-test or the Mann–Whitney test was used for post hoc testing, to ascertain which pair of groups had significantly different values.

Frequencies for binary (yes/no) variables such as hypertension, arrhythmia, vomiting in the first 24 hours, use of analgesics, or sleep disturbances after 3 months were compared between groups using the Chi-square test. Because of the high number of comparisons during data analysis, we adjusted *p* values for significance using the Bonferroni method, by dividing 0.05 by the number of comparisons [30, 31]. Therefore, *p* value for significance was adjusted to 0.05/20 = 0.0025 for the demographic and comorbidity variables presented in Table 1 and to 0.05/12 = 0.0042 for outcome variables presented in Table 2, so that only *p* values < 0.0025 or 0.0042, respectively, were considered significant. The Statistical Package for Social Sciences (IBM, SPSS statistics, version 22) was used for data analysis.

## 3. Results

**Figure 1:**
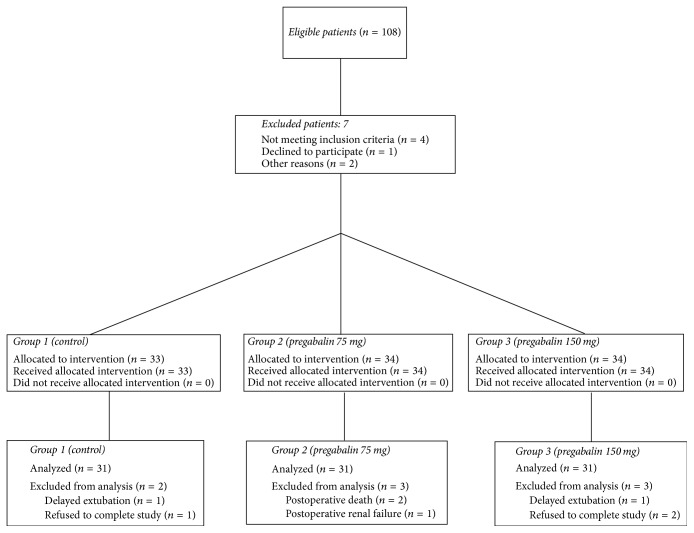
CONSORT diagram showing the progression of patients from eligibility to enrollment to completion of the study.

Of 108 eligible patients, 101 patients enrolled in the study, and 93 patients completed the study (36 women, 57 men) as shown in the CONSORT diagram in Figure 1. Demographic and clinical patient data are summarized in Table 1.

Patient age was (mean ± SD) 67.1 ± 8.3 years, weight was 80.7 ± 11.9 Kg, height was 168 ± 7 cm, and BMI was 28.5 ± 3, while median ASA physical status was 3 (range 3-4). Frequency of comorbidities and medication use initially seemed significantly different between groups for several variables. However, after adjusting the significance level to 0.05/20, therefore *p* < 0.0025 (Bonferroni correction), only duration of surgery was significantly different between groups, being significantly shorter in Group 3 (*p* = 0.002).

Data on analgesic use, adverse effects, acute postoperative pain, chronic pain, and sleep disturbances are presented in Table 2. Morphine consumption recorded from the PCA as presented in the table did not include an initial 5 mg dose given to all patients before extubation. *p* values for significance were adjusted to 0.0042 using the Bonferroni correction, as described in Methods in order to avoid false positive findings due to multiple comparisons. Differences between groups remained significant for most variables of interest even after adjusting *p* value for significance to 0.0042: the number of morphine dose requests and doses given, total morphine use in the first 8 hours and the first 24 hours, pain scores at 8 hours and at 3 months, and the frequency of sleep disturbances at 3 months were significantly different between the three groups.

When the overall testing showed a significant difference between groups for a variable of interest, as presented in Table 2, we then conducted post hoc testing using the Mann–Whitney test, in order to see which pair of groups was different with regard to each variable. Results of post hoc comparisons using the Mann–Whitney test are presented in Table 3. This table only includes variables where the overall test indicated the presence of a significant difference between groups.

With regard to vomiting, which can be very distressing for patients, there was no significant difference between groups. However, further analysis showed that morphine consumption at 8 and at 24 hours and pain scores at 8 hours and at 3 months were significantly higher in patients who experienced vomiting compared to those who did not (Table 4).

## 4. Discussion

Our results suggest that a single dose of pregabalin can result in a small but potentially beneficial reduction of acute postoperative pain and opioid consumption in the immediate postoperative period and may also reduce chronic poststernotomy pain in patients undergoing elective cardiac surgery with median sternotomy and use of cardiopulmonary bypass. Pregabalin, when added to the analgesic effect of morphine results in lower analgesia needs in the elderly, thereby leading to less severe acute postoperative pain and may potentially halt the progression to chronic pain, in accordance with the concept of “preventive analgesia” [32, 33].

Pregabalin has been used in different types of surgery and published clinical studies suggest that chronic pain may be reduced in patients who receive perioperative pregabalin [24, 26], but data on the role of pregabalin in cardiac surgery are limited: Ziyaeifard et al. [34] reported that a single preoperative dose of pregabalin 150 mg significantly reduced pain but had no effect on morphine consumption after elective CABG surgery, whereas Joshi and Jagadeesh reported that one pregabalin dose before and one daily pregabalin dose for two days after surgery reduced pain scores and tramadol consumption for 36 hours but had no effect on chronic postoperative pain in patients undergoing off-pump coronary artery bypass (OPCAB) surgery [35]. However, a prospective clinical trial by Pesonen et al. showed that although perioperative pregabalin significantly reduced perioperative oxycodone consumption and the incidence of confusion, it increased the time to extubation in elderly patients undergoing cardiac surgery [28].

Considering the age of our patients (mean age > 65 years, which is the official retirement age in many Western societies) we chose to only use a single dose of pregabalin (75 mg or 150 mg) in our study, in an attempt to minimize adverse effects in these elderly patients. Because the risk of confusion is high after cardiac surgery with cardiopulmonary bypass due to various mechanisms, including hypoperfusion, microemboli, and fast rewarming [36], we chose not to measure confusion, as we would not be able to attribute it to a specific factor. Instead, we used “number of bolus doses requested” as measure of patient agitation due to pain and “number of bolus doses given” as measure of patient analgesic needs and found that these variables differed significantly between the three groups. Although there is no obvious explanation for the observed differences between groups, it is reasonable to hypothesize that pregabalin reduces patient agitation and morphine requirement, and this effect is more pronounced with higher dose. This hypothesis is consistent with our data regarding morphine consumption at 8 hours and 24 hours after extubation where there are significant differences between all pairs of pregabalin groups.

Analysis of VRS in the first 8 hours after extubation showed significant difference between Groups 1 and 2 and Groups 1 and 3. After 24 hours the difference between Groups 1 and 2 is not significant, possibly because any difference is masked by increased morphine dose, but there is still significant difference between Groups 1 and 3. No difference is detected between Groups 2 and 3 at 8 and 24 hours. The above findings imply that a higher pregabalin dose is associated with less severe pain.

However, it is important to note that because the observed “average” differences in pain scores between groups were small, the clinical significance of such differences can be questioned. Similarly, the observed benefit with regard to reduced morphine consumption in the pregabalin groups is also small. Yet, we believe that the observed benefits with regard to pain scores and with regard to morphine consumption are clinically meaningful: differences in “maximum” scores are larger (2 points in most cases), and maximum pain is important from the viewpoint of patient experience. Similarly, maximum doses of morphine are markedly lower in patients who received pregabalin, and this reduction could be beneficial with regard to patient recovery and rehabilitation. Furthermore, it would not be realistic to expect a big, dramatic benefit from a single intervention in a complex, multifactorial care process, such as in cardiac surgery. Given the complexity of these cases, we believe that even a small reduction in pain intensity is a meaningful improvement as we all try to improve care for these patients. Three months after surgery the pattern is almost the same with patients in Group 1 experiencing more severe postoperative pain, using more analgesics and experiencing more sleep disturbances compared to patients in the pregabalin groups.

Although the small number of patients is a significant limitation of our study, the observed small but significant findings and the fact that these findings are biologically plausible and consistent with principles currently accepted in the pain literature support the validity of our findings. The observed significant difference in duration of surgery between groups, though statistically significant, is, in our opinion, of no clinical consequence and is unlikely to influence postoperative pain or any other relevant outcomes.

In conclusion, our data suggest that a single preoperative dose of oral pregabalin has small but potentially significant opioid-sparing effect, resulting in small but measurable improvement of postoperative pain with reduced morphine consumption and lower pain scores in the immediate postoperative period. The observed benefits with regard to opioid use and pain scores are small but clinically relevant, in our opinion, and may result, possibly through blockade of central sensitization mechanisms, in reduced long-term pain, with lower pain scores and fewer sleep disturbances 3 months after elective cardiac surgery. We believe that these results are truly promising. However, because this is a small, single center study and the observed clinical benefits are small, additional large, well conducted studies in different patient populations are needed to critically evaluate the validity of our findings.

## Figures and Tables

**Table 1:** Demographic and clinical patient characteristics. Data reported as mean ± SD, median (range), or number of patients, as appropriate. Values were compared between groups using ANOVA, Kruskal-Wallis, or Chi-square tests as appropriate. *p* values for significance were adjusted to 0.05/20 = 0.0025 using Bonferroni correction for multiple comparisons.

Variable	Group 1 (control) *N* = 31	Group 2 (pregabalin 75 mg) *N* = 31	Group 3 (pregabalin 150 mg) *N* = 31	*p*
Age	66.1 ± 10.2	67.4 ± 7.8	67.8 ± 6.8	0.705
Sex (M/F)	21/10	19/12	17/14	0.58
Weight	82.2 ± 14.5	80.3 ± 12.0	79.6 ± 8.9	0.665
Height	1.70 ± 0.07	1.68 ± 0.07	1.67 ± 0.07	0.342
BMI	28.6 ± 4.8	28.4 ± 3.9	28.6 ± 3.2	0.973
Operation time	243.2 ± 29.6	244.7 ± 24.2	221.9 ± 27.4	0.002
ASA status	3 (3, 4)	3 (3, 4)	3 (3, 4)	0.109
Hypertension	18	23	28	0.015
Diabetes	15	13	9	0.285
Hyperlipidemia	21	19	22	0.713
Arrhythmias	3	7	0	0.016
Thyroid disease	0	3	1	0.161
Statins	24	23	24	0.942
B-blockers	25	16	20	0.030
Ca channel antagonists	8	8	8	1.000
Diuretics	9	10	11	0.863
ACE inhibitors	10	10	7	0.625
ARBs	10	8	9	0.855
Nitrates	6	4	3	0.535
Antiplatelet agents	12	19	16	0.204

ACE = angiotensin-converting enzyme, ANOVA = Analysis of Variance, ARBs = Angiotensin Receptor Blockers, ASA = American Society of Anesthesiologists, AVR = Aortic Valve Replacement, CABG = Coronary Artery Bypass Graft, MVR = Mitral Valve Replacement.

**Table 2:** Postoperative analgesic use and pain intensity. Data reported as median (minimum, maximum). *p* values for comparisons between study groups were calculated using the Kruskal-Wallis or Chi-square tests as appropriate. *p* values for significance were adjusted to 0.05/12 = 0.0042 using Bonferroni correction for multiple comparisons.

Variable	Group 1 (control) *N* = 31	Group 2 (pregabalin 75 mg) *N* = 31	Group 3 (pregabalin 150 mg) *N* = 31	*p*
Intraoperative fentanyl (mcg)	200 (150, 350)	200 (100, 350)	200 (50, 300)	0.689
Intraoperative remifentanil (mcg)	420 (380, 600)	420 (360, 480)	400 (360, 500)	0.168
Boluses requested	12 (4, 74)	6 (0, 27)	4 (1, 26)	0.000
Boluses given	10 (4, 28)	6 (0, 14)	4 (1, 13)	0.000
VRS at 8 hours	4 (2, 6)	3 (3, 4)	3 (0, 6)	0.001
VRS at 24 hours	1 (0, 5)	1 (0, 4)	0 (0, 3)	0.007
VRS at 3 months	3 (2, 5)	2 (1, 3)	2 (1, 3)	0.000
Morphine use in first 8 hours (mg)	14 (12, 17)	13 (11, 16)	12 (11, 14)	0.000
Morphine use in first 24 hours (mg)	19.5 (16, 30)	16 (14, 22)	15 (12.5, 18)	0.000
Vomiting at 24 hours	6	4	3	0.535
Use of analgesics at 3 months	26	16	10	0.000
Sleep disturbance at 3 months	16	5	3	0.000

**Table 3:** Post hoc comparisons between groups using the Mann–Whitney test.

Groups compared	Boluses requested	Boluses given	VRS 8 hours	VRS 3 months	Morphine consumption 8 hours	Morphine consumption 24 hours	Analgesics 3 months	Sleep disturbance 3 months
1 versus 2 (control versus pregabalin 75 mg)	0.000	0.000	0.002	0.000	0.01	0.000	0.007	0.003
1 versus 3 (control versus pregabalin 150 mg)	0.000	0.000	0.002	0.000	0.000	0.000	0.000	0.000
2 versus 3 (pregabalin 75 mg versus pregabalin 150 mg)	0.003	0.004	0.465	0.985	0.1	0.005	0.126	0.452

**Table 4:** Fentanyl and morphine consumption and pain scores in patients who did versus patients who did not vomit. Data are presented as median (Min, Max). Groups were compared using the Mann–Whitney test.

Vomiting	Yes (*n* = 13)	No (*n* = 80)	*p*
Fentanyl	250 (150, 350)	200 (50, 350)	0.047
Morphine use in first 8 hours	14.0 (13.0, 16.0)	13.0 (11.0, 17.0)	0.001
Morphine use in first 24 hours	18.5 (15.0, 30.0)	16.5 (12.5, 27.5)	0.003
VRS at 8 hours	4 (4, 6)	3 (0, 6)	0.007
VRS at 24 hours	2 (0, 5)	1 (0, 4)	0.04
VRS at 3 months	3 (2, 5)	2 (1, 4)	0.005
