# Corrigendum to “Pregabalin Effect on Acute and Chronic Pain after Cardiac Surgery”

**DOI:** 10.1155/2018/5981895

**Published:** 2018-10-17

**Authors:** Aik Bouzia, Vassilios Tassoudis, Menelaos Karanikolas, George Vretzakis, Argyro Petsiti, Nikolaos Tsilimingas, Elena Arnaoutoglou

**Affiliations:** ^1^Intensive Care Unit, Medical School of Larissa, University of Thessaly, Volos, Greece; ^2^Department of Anesthesiology, Medical School of Larissa, University of Thessaly, Volos, Greece; ^3^Department of Anesthesiology, Washington University School of Medicine, St. Louis, MO, USA; ^4^Department of Cardiothoracic Surgery, Medical School of Larissa, University of Thessaly, Volos, Greece

In the article titled “Pregabalin Effect on Acute and Chronic Pain after Cardiac Surgery” [1], the presentation of the results as originally published in Table 2 can be a source of confusion for the careful reader who tries to understand these numbers, as was raised in Theodorou and Bali [2]. In order to make the presentation of these numbers more accurate, it is important to clarify that the amounts of morphine listed in the line “Morphine use in first 24 hours” do not include the amount of morphine given in the first 8 hours. Therefore, the description of the line in question should be corrected from “Morphine use in first 24 hours” to the more accurate “Morphine use in postoperative hours 9–24.” The corrected table is shown below.

## Figures and Tables

**Table 2:** Postoperative analgesic use and pain intensity. Data reported as median (minimum, maximum). Values for comparisons between study groups were calculated using the Kruskal–Wallis or chi-square tests as appropriate. Values for significance were adjusted to 0.05/12 = 0.0042 using Bonferroni correction for multiple comparisons.

Variable	Group 1 (control) *N*=31	Group 2 (pregabalin 75 mg) *N*=31	Group 3 (pregabalin 150 mg) *N*=31	
Intraoperative fentanyl (mcg)	200 (150, 350)	200 (100, 350)	200 (50, 300)	0.689
Intraoperative remifentanil (mcg)	420 (380, 600)	420 (360, 480)	400 (360, 500)	0.168
Boluses requested	12 (4, 74)	6 (0, 27)	4 (1, 26)	0.000
Boluses given	10 (4, 28)	6 (0, 14)	4 (1, 13)	0.000
VRS at 8 hours	4 (2, 6)	3 (3, 4)	3 (0, 6)	0.001
VRS at 24 hours	1 (0, 5)	1 (0, 4)	0 (0, 3)	0.007
VRS at 3 months	3 (2, 5)	2 (1, 3)	2 (1, 3)	0.000
Morphine use in first 8 hours (mg)	14 (12, 17)	13 (11, 16)	12 (11, 14)	0.000
Morphine use in postoperative hours 9–24 (mg)	19.5 (16, 30)	16 (14, 22)	15 (12.5, 18)	0.000
Vomiting at 24 hours	6	4	3	0.535
Use of analgesics at 3 months	26	16	10	0.000
Sleep disturbance at 3 months	16	5	3	0.000
